# A New Strengthening Process for Carbon-Fiber-Reinforced Thermoplastic Polyphenylene Sulfide (CFRTP-PPS) Interlayered Composite by Electron Beam Irradiation to PPS Prior to Lamination Assembly and Hot Press

**DOI:** 10.3390/ma16072823

**Published:** 2023-04-01

**Authors:** Keisuke Takeda, Hideki Kimura, Michael C. Faudree, Helmut Takahiro Uchida, Kohei Sagawa, Eiichi Miura, Michelle Salvia, Yoshitake Nishi

**Affiliations:** 1 Graduate School of Engineering, Tokai University, Hiratsuka 259-1292, Japan; 2 Graduate School of Science & Technology, Tokai University, Hiratsuka 259-1292, Japan; 3 Faculty of Liberal Arts and Science, Tokyo City University, Yokohama 224-8551, Japan; 4 Kanagawa Institute of Industrial Science and Technology (KISTEC), Ebina 243-0435, Japan; 5 Laboratory of Tribology and Dynamics of Systems (LTDS), Ecole Centrale de Lyon, 69134 Ecully, CEDEX, France

**Keywords:** composite, thermoplastic, polyphenylene sulfide, carbon fiber, interlayered, electron beam, impact strength, electron spin resonance

## Abstract

Impact by hailstone, volcanic rock, bird strike, or also dropping tools can cause damage to aircraft materials. For maximum safety, the goal is to increase Charpy impact strength (*a*_uc_) of a carbon-fiber-reinforced thermoplastic polyphenylene sulfide polymer (CFRTP-PPS) composite for potential application to commercial aircraft parts. The layup was three cross-weave CF plies alternating between four PPS plies, [PPS-CF-PPS-CF-PPS-CF-PPS], designated [PPS]_4_[CF]_3_. To strengthen, a new process for CFRP-PPS was employed applying homogeneous low voltage electron beam irradiation (HLEBI) to both sides of PPS plies prior to lamination assembly with untreated CF, followed by hot press under 4.0 MPa at 573 K for 8 min. Experimental results showed a 5 kGy HLEBI dose was at or near optimum, increasing *a*_uc_ at each accumulative probability, *P*_f_. Optical microscopy of 5 kGy sample showed a reduction in main crack width with significantly reduced CF separation and pull-out; while, scanning electron microscopy (SEM) and electron dispersive X-ray (EDS) mapping showed PPS adhering to CF. Electron spin resonance (ESR) of a 5 kGy sample indicated lengthening of PPS chains as evidenced by a reduction in dangling bond peak. It Is assumed that 5 kGy HLEBI creates strong bonds at the interface while strengthening the PPS bulk. A model is proposed to illustrate the possible strengthening mechanism.

## 1. Introduction

Carbon-fiber-reinforced polymers (CFRPs) have been increasingly utilized for commercial airplanes and space vehicles, among many other articles, due to being lightweight and having a high strength-to-weight ratio. CFs are often used for reinforcement due to their high strength, surface area, and conductivity along with inertness and stability at high temperature. Along with advanced airplane materials, potential applications of CF are: CO_2_ capture for reducing climate change [1]; battery electrodes applied to EVs [2]; and advancement of utilizations for thermally conductive carbon-reinforced composites [3]. Besides CF [4,5], several types of carbon reinforcements have been used, including: carbon nanotubes [6,7], carbon nanofibers [8], graphite, graphene, carbon black [9], and ultrathin carbon nanotube (CNT) veils to enhance interlaminar toughness [10]. 

For aerospace, a popular resin of choice has been thermoset (TS) epoxy; however, the CFRPTSs are very difficult to recycle causing serious harm to the environment if disposed of improperly, and have a long solidification time during fabrication, requiring high energy consumption. CFRP thermoplastics (CFRTPs), on the other hand, have been a viable alternative since they reduce waste by being able to be repeatedly formed and remelted, allowing recyclability to contribute to environmental sustainability. 

**Figure 1:**
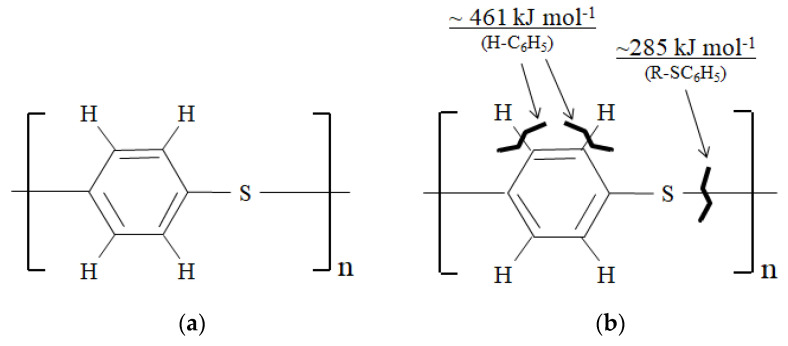
Constitutional formula of PPS: for untreated (**a**) and with reported bonding dissociation energies (BDE) [21,22] at dangling bond sites (**b**). For (**b**), ‘AR-’ is the neighboring aromatic ring (-C_6_H_5_), where the ~285 [21] and ~461 kJ mol^−1^ [22] bonds are referred to here as “AR-S” and “AR-H”, respectively.

TP resins have been increasingly used for commercial airplane parts [9,11,12], one of which is polyphenylene sulfide (PPS) with formula (C_6_H_4_S)_n_ as shown in Figure 1a. CFRTP-PPS is widely used and has application value for articles such as: ailerons, leading edge access panels, keel beam main ribs in the A340-500/600, landing flap rib in the Dornier 328, and the main landing gear door of the Fokker 50. PPS resin continues to gain attention as one of the “High Performance TPs”, similar to polyetheretherketone (PEEK) and polyetherketoneketone (PEKK) [11], that has increased rigidity due to aromatic rings inhibiting excessive backbone chain movement and increased intermolecular forces giving it strength. In PPS, sulfur (S) groups connect aromatic groups, allowing flexibility [11,13]. PPS is a widely utilized semicrystalline engineering TP polymer [9] with many advantages such as superior toughness, high modulus, tensile strength, and creep as well as excellent dimensional and high temperature stability, inherent flame resistance, and good electrical properties [4,14]. It is resistant to harsh environments [4,15] such as gasoline, oil, road salt, and exhaust gasses in high-temperature environments that airplane parts can encounter [4]. Crystallinity of PPS can reach ~60% contributing to its strength [9]. PPS has a glass transition temperature (*T*_g_) of 358 K (85 °C) and high melting temperature (*T*_m_) of ~558 K (~285 °C) [14], and can withstand higher temperatures of ~473 K (~200 °C) [11]. PPS has been used for large-scale CFRTP-PPS parts such as 3D-printed composite articles [16]; including, that of continuous CF with nominal *V*_f_ of 30 to 50%, reaching ultimate tensile strength of 1930 +/− 150 MPa [17]. CFRTP-PPS is well-researched, with recent studies that include: effect of cooling rate on elastic modulus and ultimate tensile strength [18]; heat treatment process to remove CF fabric sizing on laminates showing that Charpy impact strength was more dependent on CF volume fraction [19]; and an inventory analysis on CFRTP-PPS manufacture in the aerospace industry, exemplifying the urgent need to lower environmental impact [20].

But CFRTPs have some disadvantages, such as: (1) high processing temperature with high TP melt viscosity of 200 to 600 Pas, making flow into intricate spaces between CFs difficult [23]; and (2) TP does not adhere well to CF from their inert surfaces. Therefore, two aims of this study are: (1) to construct the laminated structure [PPS-CF-PPS-CF-PPS-CF-PPS] with alternating PPS and CF plies to minimize required melt flow distance of PPS through CF ply thickness (~230 µm) during hot press; and (2) to overcome the latter, we activate PPS plies with low voltage 100 keV-class homogeneous electron beam irradiation (HLEBI) prior to lamination assembly and hot press. 

To give a background, CFRTPs are generally weaker than CFRPTSs, mostly from poor adhesion at the CF/TP polymer interface in the form of sparse point contacts distributed heterogeneously along the CF surface [24]. As a result, treating the CF surface to strengthen the CF/TP interface has been a focus of a wide body of research, including: introduction of functional groups to the CF for increased chemical bonding [25]; electrochemical and plasma treatments [26]; electrochemical oxidation [27]; and acidic functional groups [28]. Various methods have been used to strengthen the CF/PPS interface in CFRTP-PPS composites [4,5]. In CFRTP-PPS injection-molded composites, addition of 5 wt% animated polyphenylene sulfide (PPS-NH_2_) with weight ratio (CF:PPS:PPS-NH_2_ = 20:75:5) has been found to increase tensile, flexural strength, and flexural modulus 11.4, 11.0, and 22.7%, respectively, over that without PPS-NH_2_ [4]. This was attributed to higher adhesion from the -NH_2_ groups bonding with epoxy, -C-OH, and -C-O-C- groups in the CF sizing [4]. In another study, addition of the sizing agent component carboxylic polyphenylene sulfide (PPS-COOH) to the CF surface increased interfacial shear strength of CFRTP-PPS 27 and 15% higher than untreated and plasma treated, respectively [5]. Chen, Mohanty and Misra (2021) provide a comprehensive review of carbon reinforcements in PPS composites [9].

It follows that HLEBI is an increasingly used surface treatment that does not require chemicals and can be applied to large sheets. Applying HLEBI to sample surfaces has been shown to improve fracture toughness of several CF and glass fiber (GF) FRPs [29,30,31,32,33,34,35]. Moreover, HLEBI has been utilized to directly activate CF [24,36,37,38,39,40,41] and GF [42] to increase mechanical properties of FRP. HLEBI is reported to strengthen glasses [43,44] and BMCs [33] by generating dangling bonds at low bond dissociation energy (BDE) sites with repulsive force between electrons, creating internal compressive forces. In CF [28] and PPS, HLEBI decreases dangling bond density, probably due to 6-membered rings of conjugated carbon atoms. However, a novelty of this study is that we use HLEBI to treat the matrix of PPS, not the CFs. Figure 1b shows sites in PPS where BDE of aromatic-S (AR-S) bond is much lower (~285 kJmol^−1^) than AR-H at 461 kJmol^−1^ [14]. Since monomers are connected by sulfide (-S-) groups, reducing AR-S dangling bonds would assumably lengthen PPS chains, strengthening the PPS structure. Moreover, charge transfer would occur from the activated PPS to the CFs, enhancing adhesion at the CF/PPS interface and strengthening the interlayered [PPS]_4_[CF]_3_ composite. 

Up to now, there has been few studies of HLEBI to strengthen CFRTP-PPS composites. One study was found applying 32 to 90 kGy HLEBI to CFRTP-PPS; however, data appear inconclusive due to few data points [45]. Therefore, the goal of this study is to demonstrate that, for a [PPS]_4_[CF]_3_ interlayered composite of three CF plies alternated between four PPS plies [PPS-CF-PPS-CF-PPS-CF-PPS], the new process of applying HLEBI to PPS plies prior to lamination assembly with untreated CF plies and hot press can increase the important mechanical property of impact strength for potential application to airplane parts. A model is proposed to explain the strengthening mechanism by HLEBI in PPS plies themselves, and the CF/PPS interface.

## 2. Experimental Procedure

### 2.1. Preparation of Sized CF and PPS 

Plain cross-weave CF (TR3110M) plies from Mitsubishi Rayon Ltd., Tokyo, Japan were used with listed areal weight of 198 to 200 gm^−2^, and nominal thickness of 230 µm [46]. The CFs were provided with typical epoxy film sizing coating with nano-thickness and whose composition was determined by proton-NMR (AVANCE500, Neutron Magnetic Resonance, Shimazu, Kyoto) [47]. Branched type PPS was used (B-063S, TOSO Co. Ltd., Tokyo, Japan) that has been utilized for automobile parts such as gears, fuel, and other fluid transport tubes and ducts, along with electrical systems. Branched type PPS was chosen because it is reported to have higher molecular weight (M.W.) and better mechanical properties such as higher ductility than that of normal PPS which has a M.W. of ~18,000 [14]. Specific mechanical and other properties of the provided PPS are not listed and are proprietary. 

### 2.2. Composite Fabrication

**Figure 2:**
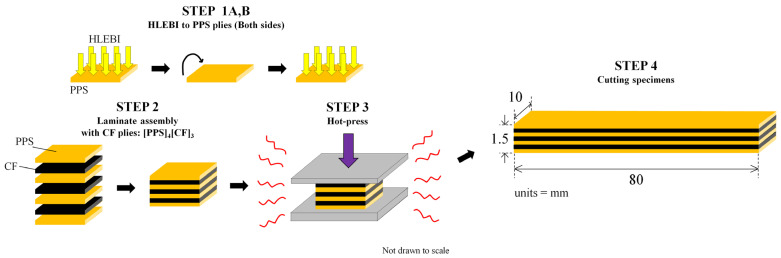
Fabrication steps for HLEBI-treated [PPS]_4_[CF]_3_ interlayered composite.

CFRTP-PPS composite fabrication consisted of four basic steps as shown in Figure 2: 

**Step 1:** HLEBI was applied homogeneously to both side surfaces of PPS sheets (see the next section). Step 1 is eliminated for untreated samples.

**Step 2:** Laminate assembly was carried out with 3 plies of sized untreated CF placed alternately between 4 plies of PPS to obtain a lay-up of [PPS-CF-PPS-CF-PPS-CF-PPS], designated here as [PPS]_4_[CF]_3_.

**Step 3:** Samples were then cured by one-directional hot press (IMC-185A, Imoto Machinery Co., Ltd., Tokyo, Japan) at 4.0 MPa and 573 K for 8 min. 

**Step 4:** Samples were cut to size: length, width, and thickness of 80 × 10 × 1.5 mm.

The HLEBI samples were compared to a control without HLEBI (eliminating Step 1). Volume fraction, *V*_f_ of CFs in the laminate samples was 0.55 (55%). Nine samples each were tested for each data set of: untreated, 5, 10, 20, and 30 kGy HLEBI conditions. 

### 2.3. Conditions of HLEBI

**Figure 3:**
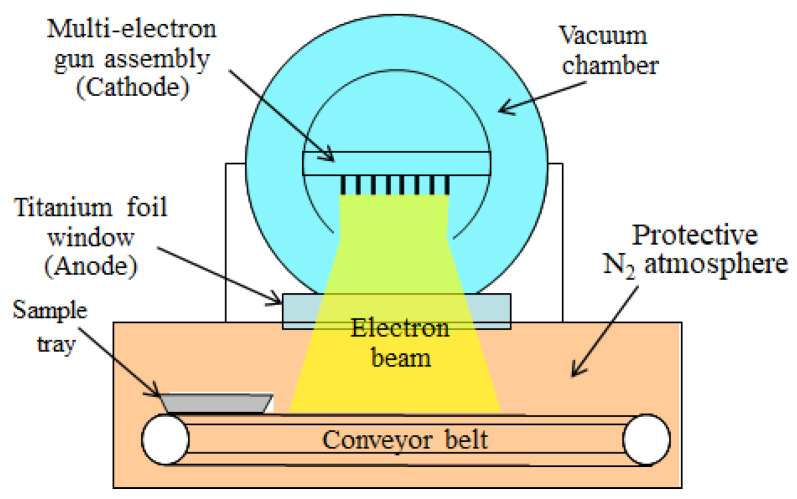
Schematic diagram of electron curtain processer. (Iwasaki Electric Group Co., Ltd., Tokyo, Japan).

PPS plies were treated by HLEBI on both sides by an electron–curtain processor (Type CB175/15/180L, Energy Science Inc., Woburn, MA, USA, Iwasaki Electric Group Co., Ltd., Tokyo, Japan) prior to lamination assembly with untreated sized CFs. Acceleration voltage, and distance between sample and Ti thin film window were 170 kV and 25 mm, respectively. Figure 3 shows a schematic of the electron curtain processor. HLEBI setup and parameters are described in detail in [38]. 

Based on the density of PPS (1350 kgm^−3^) [48], electron beam penetration depth, *D*_th_, is calculated to be 164 μm [49]. Within *D*_th_, dangling bonds (Figure 1b) are reported to be formed [29,33,42] or reduced [29,36,37] depending on the material treated. During lamination assembly and hot press, charge transfer should occur from PPS into the highly conductive CF. Note HLEBI was applied to PPS plies only, not CF plies.

### 2.4. Charpy Impact Test

**Figure 4:**
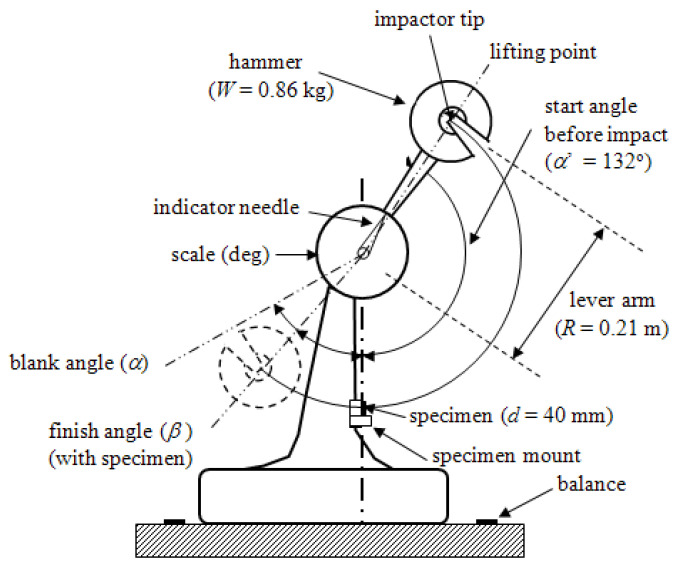
Schematic of the Charpy impact machine (Shimadzu Corporation No.51735, Tokyo, Japan) taken from Faudree, Nishi, Gruskiewicz, Salvia (2018) [51]. Angles *α α*νδ *β* are exaggerated for clarity.

The Charpy impact test is typically used to give a rough or better estimation to screen candidate airplane materials for further testing such as impact drop tower, compression after impact (CAI), edge delamination strength (EDS), and tensile, to name a few. Charpy impact tests were carried out using a standard impact fracture energy measurement system (Shimadzu Corporation No.51735) in accordance with Japanese Industrial Standard, (JIS K 7077) [20,36,50]. Figure 4 illustrates a schematic. Impact fracture energy, *E* (kJ) is calculated by Equation (1) [32,50]:

*E* = *WR*[(cos*β*-cos*α*) − (cos*α*′-cos*α*)][(*α* + *β*)/(*α-α′*)] (1)

Here, *E*, *W*, *R*, *β*, *α*, and *α′* are: impact fracture energy (kJ); hammer mass (kg); length (m) of hammer weight point from rolling center; maximum angle after impact (Radians); start angle before impact (*a* = 2.3 Radians or 132°); and maximum angle of blank test, respectively. Angles are read by mechanical indicator needle in Figure 4.

Three blank tests are conducted to calibrate the impactor for environmental conditions such as atmospheric pressure, temperature, and humidity. Charpy impact strength (kJ m^−2^) is calculated by Equation (2):

*a_uc_* = *E*/(*bt*) (2)

Here, *E, b* (=10 ± 0.2 mm) and *t* (=1.5 ± 0.15 mm) are: impact fracture energy (J), sample width (mm), and thickness (mm). The distance *d* between supporting points in the specimen holder was 40 mm. 

### 2.5. Accumulative Probability

Accumulative probability (*P*_f_) is a statistical calculation to rank samples from weakest to strongest, assigning numeric strength between 0.0 and 1.0. *P*_f_ is calculated in Equation (3) based on the median rank method [52]:

*P*_f_ = (*i −* 0.3)/(*N*_s_ + 0.4) (3)

Here, *N*_s_ and *i* are the total number of samples (9) and rank order integer of Charpy impact strength of each sample (1 to 9). For *i* of 1, 5, and 9, *P*_f_ are 0.07, 0.50, and 0.93, respectively.

### 2.6. Microscopy and Energy Dispersive Spectroscopy (EDS)

To examine sample surfaces, an optical microscope (12.7: 1 optical zoom ratio, Nikon Model SMZ1270i, Tokyo, Japan) was used, along with a JEOL SEM (Model JCM-6000PLUS, Tokyo, Japan) with EDS to obtain elemental mapping (acceleration voltage 10 kV; irradiation current 7.47500 nA). 

### 2.7. Electron Spin Resonance (ESR) Spectroscopy

To detect dangling bonds in PPS before and after HLEBI, PPS samples were analyzed by an electron spin resonance spectrometer (ESR, JES-FA2000, Nippon Denshi, Ltd., Tokyo, Japan). ESR detects spins of unpaired electrons (*m*_s_ = +/−1/2) since electrons have spin quantum number and magnetic moment. Magnetic moments of the unpaired electrons align themselves parallel or antiparallel to an applied magnetic field, resulting in output peak at a specific magnetic field, *B* [33]. 

## 3. Results

### 3.1. Relationship between HLEBI to PPS and Impact Strength of [PPS]_4_[CF]_3_ Samples

**Figure 5:**
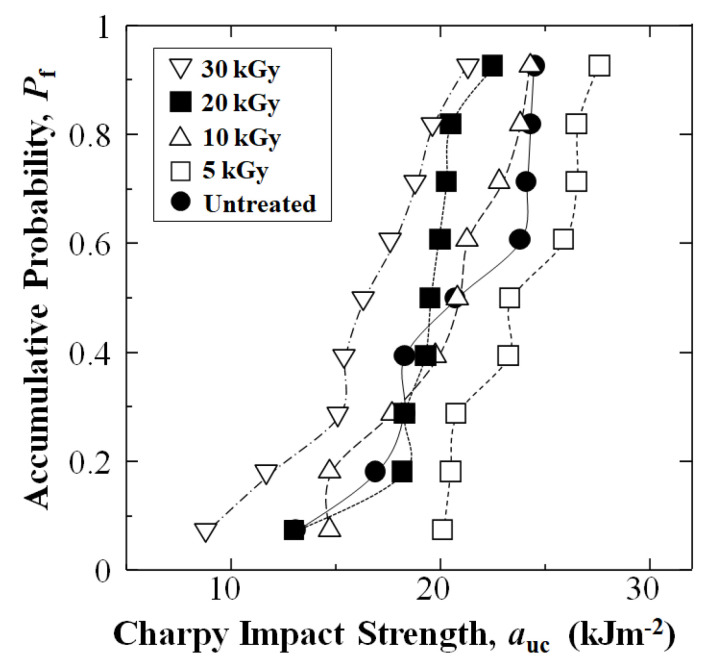
Plot of accumulative probability, *P*_f_ vs. Charpy impact strength, *a*_uc_ (kJ m^−2^) as a function of HLEBI dose.

Figure 5 and Table 1 show experimental results of changes in Charpy impact strength (*a*_uc_) of the CFRTP-PPS [PPS]_4_[CF]_3_ composite as a function of accumulative probability (*P*_f_) for data sets of untreated and HLEBI treated of 5, 10, 20, and 30 kGy, respectively. Namely, the small dose of 5 kGy appears to be at or near the optimum for improving impact resistance since it raised *a*_uc_ at each *P*_f_. Importantly, the 5 kGy dose increased *a*_uc_ significantly (53%) for the weakest samples in the datasets (*P*_f_ = 0.07) from 13.1 to 20.1 kJ m^−2^, indicating increased reliability and safety. 

However, Figure 5 and Table 1 show that as the HLEBI dose was increased from 10 to 30 kGy the *a*_uc_ was decreased; the 30 kGy resulting in the lowest *a*_uc_ due to excess radiation damage. 

**Figure 6:**
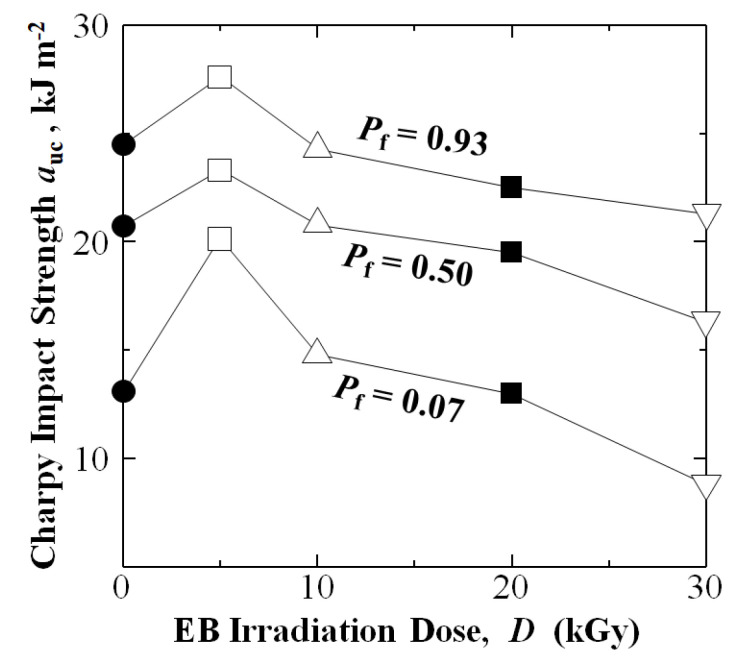
Effect of HLEBI irradiation dose on experimental impact strength (*a*_uc_) at low- (*P*_f_ = 0.07), median- (0.50), and high- (0.93) fracture probabilities for untreated and HLEBI-irradiated [PPS]_4_[CF]_3_ samples, respectively.

Figure 6 plots *a*_uc_ of [PPS]_4_[CF]_3_ for low-, median-, and high-*P*_f_ of 0.07, 0.50, and 0.93, respectively, showing *a*_uc_ at maximum at 5 kGy, then decreasing as the dose is increased from 10 to 30 kGy. The 5 kGy HLEBI dose increased *a*_uc_ to 20.1, 23.3, and 27.6 kJ m^−2^, respectively, which are 53%, 12%, and 13% higher than those untreated samples at 13.1, 20.7, and 24.5 kJ m^−2^ (Figure 6 and Table 1). In contrast, the 10 kGy dose increased *a*_uc_ slightly at low-*P*_f_ of 0.07 from 13.1 to 14.7 kJ m^−2^; while, resulting in slight to no change compared to untreated samples at *P*_f_ = 0.50 from 20.7 to 20.8 kJ m^−2^ and at *P*_f_ = 0.93 from 24.5 to 24.3 kJ m^−2^. 

The 20 kGy reduced *a*_uc_ to 13.0, 19.5, and 22.5 kJ m^−2^; and 30 kGy lowered *a*_uc_ further to 8.9, 16.3, and 21.3 kJ m^−2^ at *P*_f_ of 0.07, 0.50, and 0.93, respectively. 

### 3.2. Determination of Statistically Lowest Impact Strength, a_s_ (a_uc_ at P_f_ = 0)

The statistically lowest impact strength, *a*_s_ (*a*_uc_ at *P*_f_ = 0), is calculated by the 3-dimensional Weibull calculation [53] for each data set. In industry, for a batch of manufactured products, the *a*_s_ calculation is commonly used to determine the statistically weakest part to estimate safety limits and reliability in quality control (QC). 

When the equation is assumed to be applicable to the experimental *a*_uc_, the *P*_f_ depends on risk of fracture [32,53]. The *a*_s_, coefficient *m*, and the constant *a*_III_, are key parameters for predicting the required strength for new structural materials,

*P*_f_ = 1 − exp[–([*a*_uc_ − *a*_s_]/*a*_III_)^m^] (4)

with linear form:

ln(−ln(1 − *P*_f_)) = *m*ln(*a*_uc_ − *a*_s_) − *m*ln*a*_III_ (5)

where *m* is shape parameter, and *a*_III_ is scale parameter or characteristic strength [54]. 

**Figure 7:**
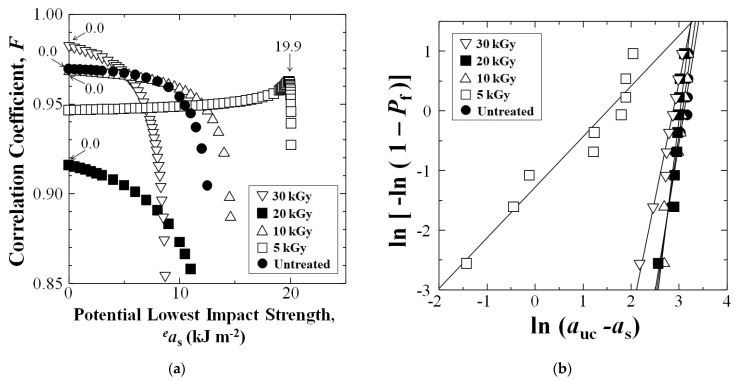
Iteration of potential lowest imact strength (*^e^a_s_*) to obtain statistically lowest impact strength *a_s_* (*a*_uc_ at *P*_f_ = 0) when correlation coefficient *F* reaches the maximum (arrows) (**a**); and linear relationships between ln(*a*_uc_ − *a*_s_) and ln[-ln(1 − *P*_f_)] from the 3-dimensional Weibull calculation (**b**).

Figure 7a shows when linear form Equation (5) is iterated to the highest correlation coefficient *F*, the *a*_s_ is obtained. Iteration is done with Microsoft Excel 97-2003 inputting potential lowest impact values ^e^*a*_s_ until *F* is at a maximum. The 5 kGy HLEBI data set (squares) exhibited the highest *a*_s_ at 19.9 kJ m^−2^, indicating that the 5 kGy HLEBI increases safety and reliability of [PPS]_4_[CF]_3_ samples. Figure 7b shows linear plots between ln (*a*_uc_ − *a*_s_) and ln [−ln(1 − *P*_f_)] whose slopes and *y*-intercepts are *m* and − *m*ln*a*_III_, respectively. 

Average *a*_uc_ and standard deviations (in brackets) for untreated, 5, 10, 20, and 30 kGy HLEBI data sets are 20.4 (4.1), 23.8 (2.9), 20.0 (3.6), 19.1 (2.6), and 16.1 (3.9) kJ m^−2^, respectively. However, the focus here is plotting *P*_f_ vs. *a*_uc_, as shown in Figure 5, since it indicates type of scatter. Standard deviation does not indicate if some specimens have much lower *a*_uc_ than the rest, or if the bulk of scatter is in stronger specimens. Therefore, Figure 5 and Figure 7a clearly show that the 5 kGy HLEBI improved *a*_uc_ at all *P*_f_ over untreated; and the *a*_s_ (*a*_uc_ at *P*_f_ = 0) of 19.9 kJ m^−2^ is the maximum over all other data sets, which exhibited *a*_s_ of 0 kJ m^−2^. 

### 3.3. Optical Microscopy Observation

**Figure 8:**
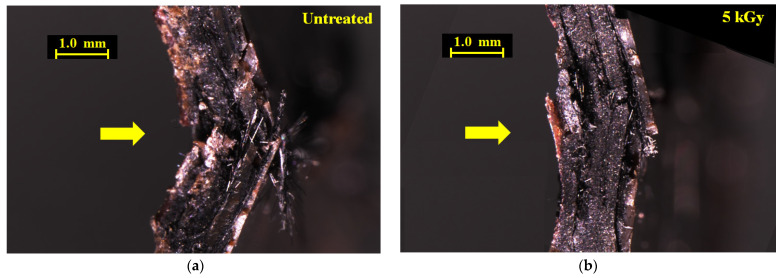
Optical microscope photos of damage zones (side view) of untreated (**a**) and 5 kGy (**b**) [PPS]_4[_CF]_3_ samples, respectively. Arrows show Charpy impact direction.

To explain strengthening of the [PPS]_4_[CF]_3_ samples by HLEBI, Figure 8a,b show optical microscopy photos of impacted untreated and 5 kGy [PPS]_4_[CF]_3_ samples, respectively. Side views are shown, arrows indicating impact direction. Most evident is that plies of the 5 kGy sample maintained cohesion within the interlayered structure with little or no CF separation or pullout compared with untreated samples. The 5 kGy sample is bent to a much lower degree, indicating increased rigidity in the interlayered system. On the other hand, the untreated sample shows ply separation with CFs protruding out from the tensile surface of the impact zone, and a high degree of damage within its interlayered structure. 

**Figure 9:**
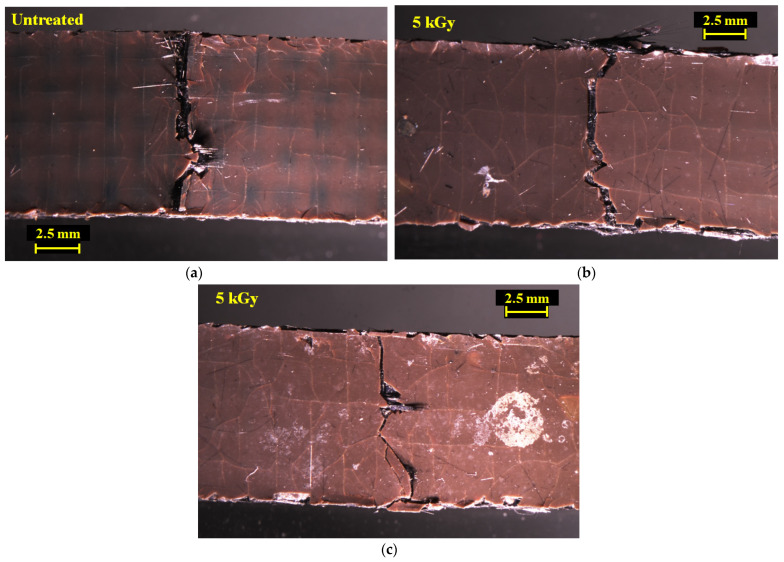
Optical microscope photos of tensile sides of untreated (**a**) and 5 kGy [PPS]_4[_CF]_3_ samples (**b,c**), respectively.

**Figure 10:**
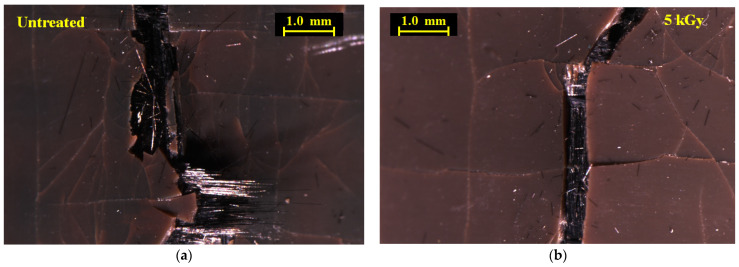
Optical microscope photos of tensile side of untreated (**a**) and 5 kGy (**b**) [PPS]_4_[CF]_3_ samples, respectively, showing a close-up of the main crack.

Figure 9a–c show photos of the tensile side surface for untreated and two 5 kGy samples. Notably, Figure 9 shows that the main cracks across the outer PPS ply of the 5 kGy samples are narrower than those of untreated samples. Here, little or no CFs are seen projecting from the main crack, although some CFs are projecting from the specimen side (top) in Figure 9b. Figure 10a,b show closeups from Figure 9a,b. Most evident is in the 5 kGy sample, where the CF ply under the outer PPS ply exhibits consolidation with no CF separation observed and the CF cross-weave can be clearly seen. In contrast, in untreated sample CF plies, under the main crack are damages with separation and pull-out. The 5 kGy HLEBI apparently prevented the main crack from propagating from the outer PPS ply into the adjoining CF ply. In summary, Figure 8, Figure 9 and Figure 10 indicate increased adhesion at the CF/PPS interface as the 5 kGy HLEBI leads to increased resistance to CF pull-out and improved impact strength. 

### 3.4. SEM and EDS Observation

**Figure 11:**
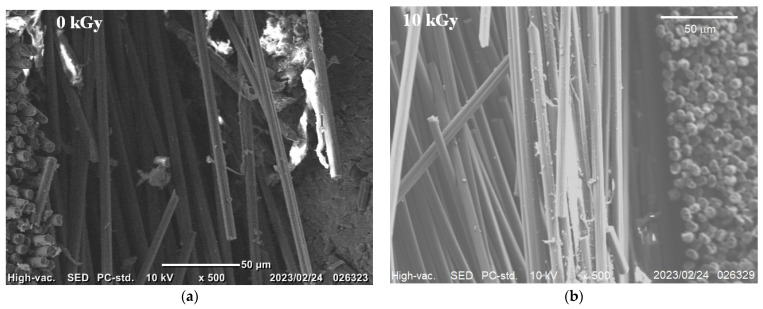
SEM photomicrographs of untreated (0 kGy) (**a**) and 10 kGy (**b**) [PPS]_4_[CF]_3_ samples, respectively.

To explain damage reduction in Figure 8, Figure 9 and Figure 10 by HLEBI, SEM, and EDS, analyses were carried out. Figure 11 shows SEM photomicrographs of untreated (a) and 10 kGy (b) fracture surfaces. Figure 10b shows that HLEBI increases PPS/CF adhesion, as evidenced by PPS adhering to CF at point contacts, although it could not be found in the untreated sample. 

**Figure 12:**
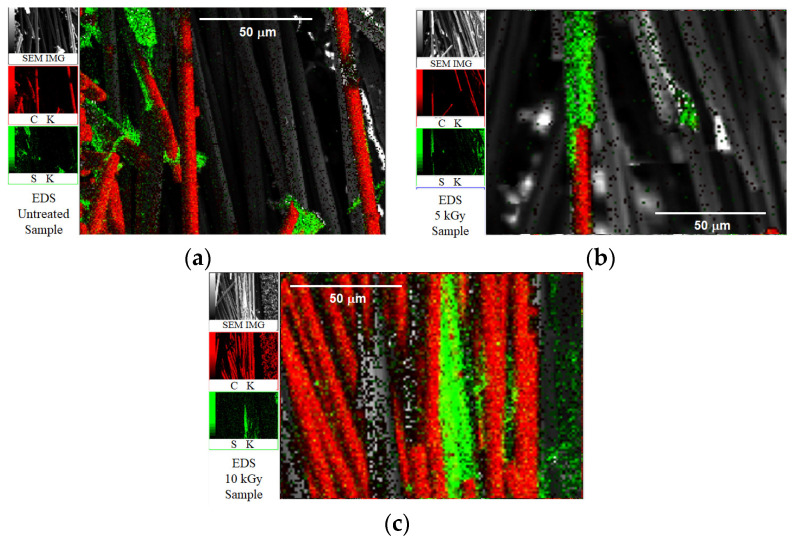
EDS mapping of fracture surfaces of untreated (**a**) 5 kGy (**b**) and 10 kGy (**c**) [PPS]_4_[CF]_3_ samples, respectively.

Figure 12 shows EDS element mappings for untreated (a), 5 kGy (b), and 10 kGy (c) samples, respectively, where red and green represent oxygen (O) Kα and sulfur (S) Kα and Kβ emissions. Here, S represents PPS resin. The untreated sample shows clean CF surfaces, whereas the 5 kGy apparently shows retention of S (PPS) on the CF extending with the CF shape. In addition, the 10 kGy indicates PPS adhering to and spanning between CFs, along with point contacts on the CFs. Overall, Figure 11 and Figure 12 show that HLEBI can increase PPS/CF adhesion. However, while the 10 kGy dose increases PPS/CF adhesion, it lowers *a*_uc_ below that of 5 kGy by excess radiation damage which is described in the next section.

### 3.5. Increasing Fiber Pull-Out Resistance and ESR Results

As mentioned earlier, HLEBI is reported to increase fiber pull-out resistance between CF and TPs in CFRTP-PEEK [55], CFRTP-ABS [41], and CFRTP-PC [40]. In short fiber CFRTP-PEEK, SEM observation of fracture surfaces showed that HLEBI increases the area of PEEK adhering to CF, with PEEK spanning between CFs for more consolidated structure [55].

To characterize the strengthening mechanism on the molecular scale, ESR analysis of untreated and HLEBI-treated PPS plies was carried out. This is because ESR has been widely implemented as a tool to characterize reaction mechanisms in polymer systems detecting free radicals during chain growth, or depolymerization reactions [56]. Dangling bonds are free radicals that are typically immobile [56].

**Figure 13:**
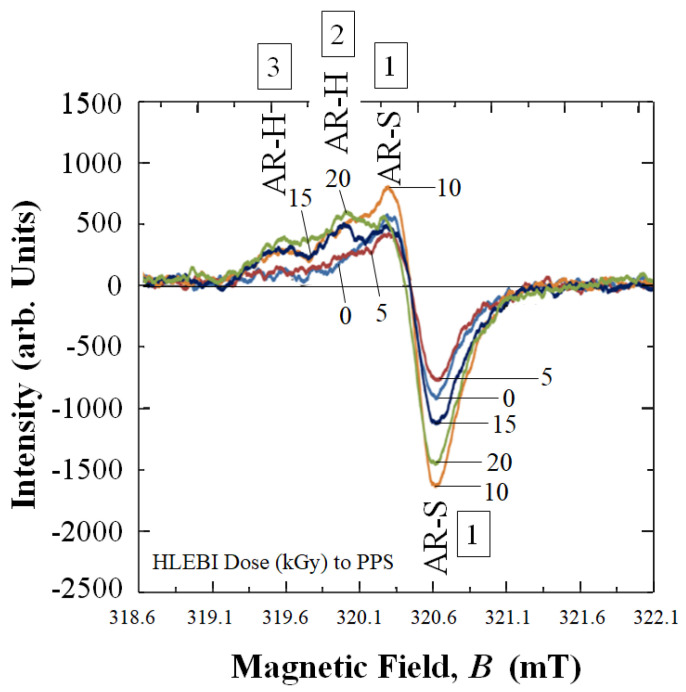
ESR signals of untreated samples (0 kGy) and those with the HLEBI dose (kGy) to PPS. Peaks 1, 2, and 3 corresponding to dangling bonds are indicated.

Figure 13 shows experimental results of ESR analysis of PPS untreated, along with HLEBI treated at 5, 10, 15, and 20 kGy. Table 2 shows a summary. Based on BDE, intensity change in ESR signals of PPS in Figure 13 can be explained. Three peaks were detected, labelled “1”, 2”, and “3”: Peak 1 being the large peak whose inflection point is at magnetic field *B* at 320.3 mT. Although ESR peaks cannot generally determine the kind of dangling bonds, *B* of Peak 1 at 320.3 mT is assumed to represent spontaneous AR-S dangling bonds; while, Peaks 2 and 3 at *B* = 319.9 and 319.5 mT are assumed to represent AR-H. This is because AR-S has significantly lower BDE of 285 kJ mol^−1^ compared with AR-H at 461 kJ mol^−1^ [22]. Note both Peaks 2 and 3 only appear at higher HLEBI treatments of 10 kGy and above, and the Peak 2 is higher intensity than Peak 3.

Figure 13 shows that Peak 1 was reduced by 5 kGy HLEBI, indicating decreasing AR-S dangling bond density. Since ESR detects free radicals, the mechanism is assumed to be chain lengthening to strengthen the PPS matrix. In addition, the heat energy of HLEBI apparently acted to recover the AR-S bonds. Moreover, upon lamination assembly and hot press, charge would transfer to the CF/PPS interface and into the highly conductive CF, enhancing adhesion by free radicals bonding to the CF and its sizing. 

However, Peaks 2 and 3 in Figure 13 at *B* = 319.9 and 319.5 mT, without inflection points, probably represent AR-H dangling bonds. Spontaneous dangling bonds cannot be found; the BDE of AR-H (461 kJ mol^−1^) being 64% higher than that of AR-S (285 kJ mol^−1^) [21]. As shown in Figure 13, spontaneous AR-H dangling bonds do not appear to occur naturally in untreated PPS, as evidenced by absence of Peaks 2 and 3 at 319.9 and 319.5 mT. At the low dfose of 5 kGy, the Peaks 2 and 3 were not detected. However, since higher HLEBI doses of 10 to 20 kGy-HLEBI increase intensities of Peaks 2 and 3, they probably increase AR-H dangling bond density. 

On the contrary, the higher dose of 10 kGy HLEBI resulted in an AR-S peak at maximum intensity, indicating maximum AR-S dangling bond density. This indicates an increase in free radicals [56], apparently shortening PPS chains and lowering molecular weight of the PPS, acting to lower the *a*_uc_. Likewise, 15 and 20 kGy peaks were higher than untreated and 5 kGy samples. Conversely, AR-H dangling bonds were generated as shown by the appearance of Peaks 2 and 3 only at higher doses of 10, 15, and 20 kGy. This may have also acted to weaken the *a*_uc_.

Table 2 summarizes relationship between HLEBI dose, AR-S, AR-H dangling bonds, and *a*_uc_ at *P*_f_ = 0.50. 

## 4. Discussion

### 4.1. Dangling Bond Formation 

**Figure 14:**
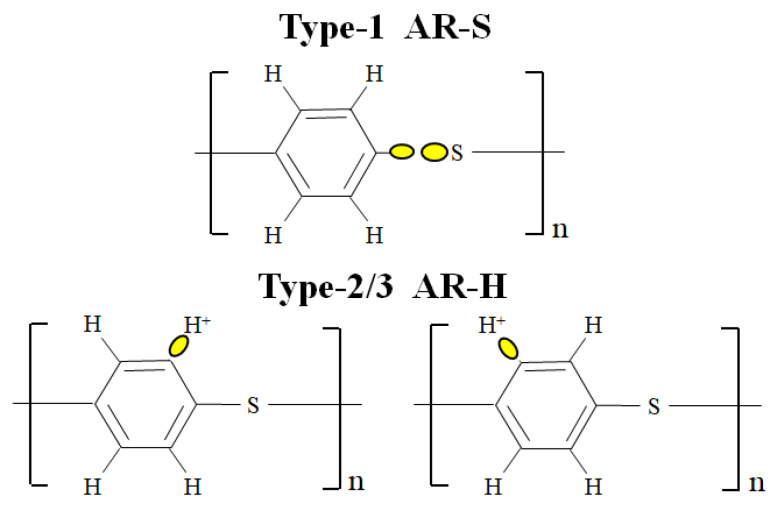
Constitutional formula of PPS with three types of dangling bonds assumed from Figure 13.

Figure 14 illustrates the three types of dangling bonds in PPS: AR-S designated “Type 1”; with AR-H designated “Type 2” and “Type 3”. Types 1, 2, and 3 refer to Peaks 1, 2, and 3 in Figure 13. Types 2 and 3 are regarded as identical, having identical location within the PPS macromolecule. The differences in *B* = 319.9 and 319.5 mT and their intensities in Figure 13 may be due to slightly different electrical configurations in the vicinity of the AR-H dangling bonds, brought about by movement probabilities between PPS macromolecules or within PPS macromolecules themselves, which is beyond the scope of this study. Henceforth, both Types 2 and 3 of AR-H will be referred to here as “Type 2/3” dangling bonds. 

### 4.2. Model of Proposed Strengthening Mechanism by HLEBI in PPS Plies and at CF-PPS Interface 

**Figure 15:**
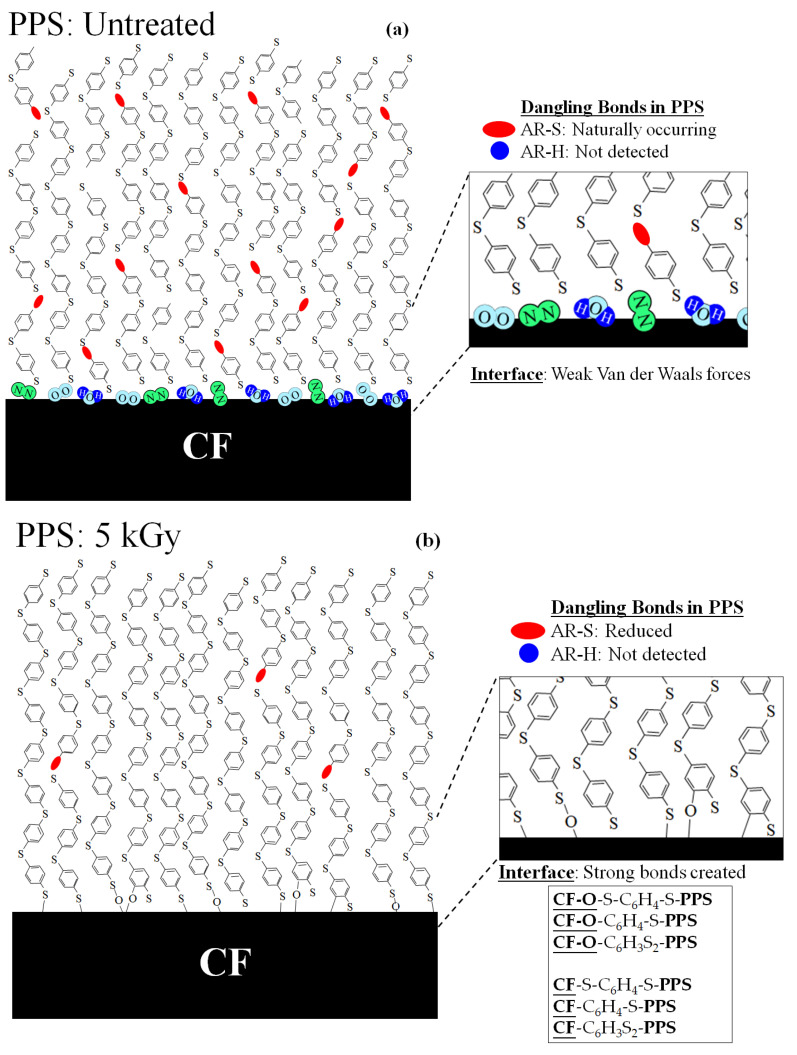

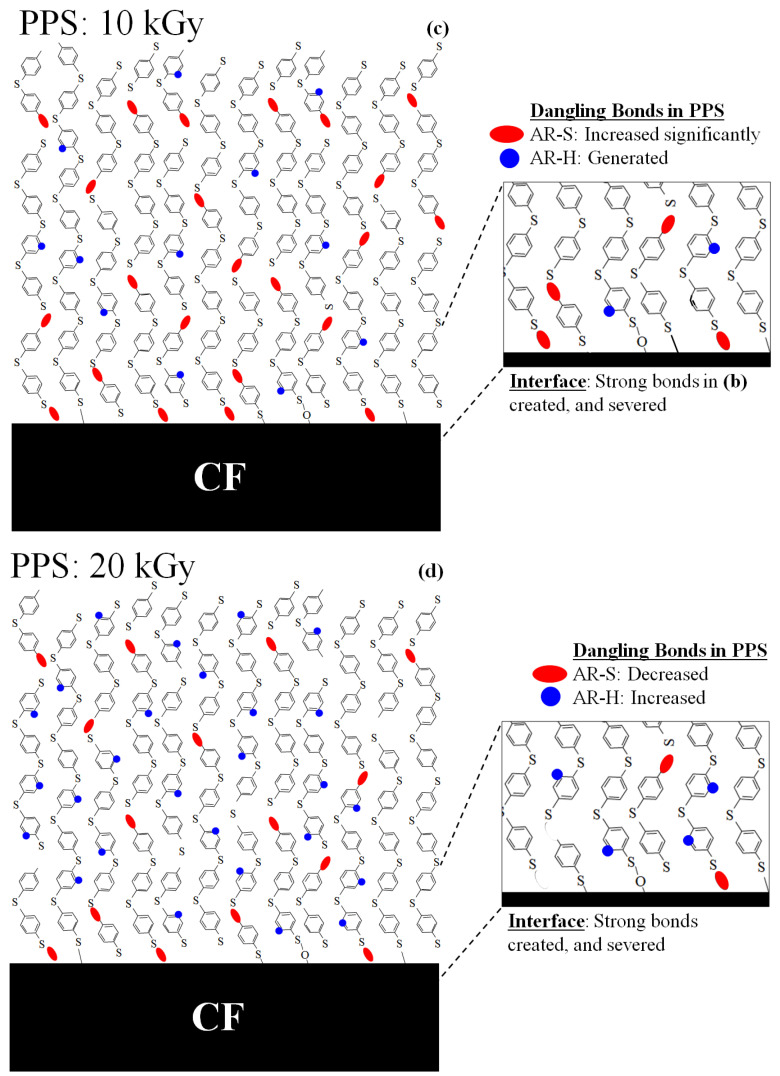
Proposed model of a strengthening mechanism by HLEBI within the PPS matrix and at the CF/PPS interface for: (**a**) untreated, (**b**) 5 kGy, (**c**) 10 kGy, and (**d**) 20 kGy [PPS]_4_[CF]_3_ samples, respectively. AR-S and AR-H dangling bonds are modelled as red ellipses and blue dots, respectively. Note that the number of bonds shown is arbitrary; the purpose is just to model trends by the HLEBI.

Figure 15 illustrates a molecular model of a proposed strengthening mechanism for [PPS]_4_[CF]_3_ by HLEBI in the PPS matrix and at CF/PPS interface. When Type-1 and Type 2/3 dangling bonds (Figure 14) correspond to 1st, 2nd, and 3rd ESR peaks (Figure 13), respectively, the strengthening mechanism of [PPS]_4_[CF]_3_ composite can be explained. 

**PPS: Untreated**

Figure 15a illustrates untreated PPS with naturally occurring AR-S dangling bonds (ellipses) with no AR-H dangling bonds (dots). At the interface, weak Van der Waals forces from H_2_O, O_2_, and N_2_ gas molecules creating some adhesions are depicted. 

**PPS: 5 kGy**

Figure 15b illustrates a reduced number of Type 1 AR-S dangling bonds in PPS by the 5 kGy HLEBI, with AR-H dangling bonds as non-existent. Decreased AR-S dangling bonds are assumed to be chain lengthening to strengthen the PPS matrix. In addition, at the interface, 5 kGy HLEBI is assumed to generate strong bonds, possibly covalent, from charge transfer from activated PPS to the highly conductive CF. Bonding of PPS is assumed to be with oxygen (O) in CF sizing or CF itself. Examples would be: **CF-O**-S-C_6_H_4_-S-**PPS, CF-O**-C_6_H_4_-S-**PPS**, and **CF-O**-C_6_H_3_S_2_-**PPS**; and with CF itself as **CF**-S-C_6_H_4_-S-**PPS, CF**-C_6_H_4_-S-**PPS,** and **CF**-C_6_H_3_S_2_-**PPS**, where “**CF-O**” is sizing, “**CF**” is CF surface, and “-C_6_H_x_-“ is -AR-.

As mentioned earlier, similar to PPS, CF has naturally occurring dangling bonds that are reduced by HLEBI [30]. When HLEBI is directly applied to CFs, it has been reported to raise bending fracture strain [43,57], along with deformation resistivity, tensile strength, and strain of CF itself [44]. Since this study was with HLEBI applied to PPS only, it can be assumed that charge transfer occurs from PPS to CF to enhance the CF as well, to collectively strengthen the [PPS]_4_CF]_3_ composite. 

**PPS: 10 kGy**

Figure 15c illustrates for the 10 kGy dose, increased AR-S and the appearance of AR-H dangling bond densities. The increased AR-S dangling bonds are apparently PPS chains being shortened. This, together with AR-H generation, apparently weakens the PPS matrix. In addition, at the CF/PPS interface, strong bonds are apparently created but also severed by the excess HLEBI dose; the net number is apparently reduced from that at 5 kGy. Therefore, the 10 kGy dose reduces *a*_uc_. 

**PPS: 20 kGy**

Figure 15d illustrates decreased AR-S dangling bonds at a 20 kGy dose compared to the 10 kGy, but still higher than untreated samples, again indicating shortened PPS chains. This, accompanied by an increase in AR-H dangling bonds, is assumed to weaken the PPS bulk below that at 10 kGy. At the CF/PPS interface, a higher number of strong bonds are apparently severed by the excess HLEBI dose, reducing *a*_uc_ below that at 10 kGy. At a higher dose of 30 kGy, it is assumed that AR-S and AR-H dangling bonds would be higher, since *a*_uc_ is reduced further. 

Overall, Figure 15 illustrates the 5 kGy HLEBI dose apparently works best to strengthen the PPS itself in concert with the CF/PPS interface, increasing *a*_uc_ of the multilayered [PPS]_4_[CF]_3_ composite.

## 5. Conclusions

Since impacts such as hailstone, volcanic rock, bird strike, or also dropping tools can damage aircraft materials, a new strengthening process for an interlayered composite typically used in aerospace of carbon-fiber-reinforced thermoplastic polyphenylene sulfide polymer (CFRTP-PPS) was proposed to raise impact strength. This research aims to have application for commercial aircraft parts. Experimental results showed that when homogeneous low voltage electron beam irradiation (HLEBI) was applied to both sides of PPS plies prior to lamination assembly with untreated CF plies, and hot press, Charpy impact strength was increased. Samples were three CF plies alternating between four PPS plies, [PPS-CF-PPS-CF-PPS-CF-PPS] designated as [PPS]_4_[CF]_3_.

(1) Applying 5 kGy-HLEBI to PPS improved Charpy impact strength (*a*_uc_) at accumulative fracture probabilities *P*_f_ of 0.07, 0.50, and 0.93: from 13.1, 20.7, and 24.5 kJ m^−2^ to 20.1, 23.3, and 27.6 kJ m^−2^, respectively; increases of 53%, 12%, and 13%. The *a*_uc_ was improved most at the low-*P*_f_ of 0.07 (53%), indicating increased reliability by strengthening of the weakest samples in the data set.
(2) The 3-dimensional Weibull analysis, often used for QC, showed the 5 kGy-HLEBI data set exhibited the highest *a*_s_ at *P*_f_ = 0 at 19.9 kJ m^−2^, indicating an increase in safety and reliability of the 5 kGy [PPS]_4_[CF]_3_ samples.
(3) Optical microscopy along with SEM and EDS showed the 5 kGy HLEBI dose increased PPS/CF adhesion and increased cohesion within the interlayered structure to raise the impact strength.
(4) A model was constructed to explain strengthening of PPS plies themselves and increased adhesion at the CF/PPS interface. (1) In the PPS matrix, reduced AR-S dangling bond density (which exists naturally in untreated PPS) acts to lengthen the chains as evidenced by a reduction in ESR peak with an inflection point at 320.3 mT. (2) At the CF/PPS interface, strong bonding is maximized in the form of **CF-O**-S-C_6_H_4_-S-**PPS, CF-O**-C_6_H_4_-S-**PPS** and **CF-O**-C_6_H_3_S_2_-**PPS**; and with CF itself, as **CF**-S-C_6_H_4_-S-**PPS, CF**-C_6_H_4_-S-**PPS** and **CF**-C_6_H_3_S_2_-**PPS**, where “**CF-O**” is sizing, “**CF**” is CF surface, and “-C_6_H_x_-“ = -AR-. On the CF surface, EDS results detected sulfur, while SEM detected PPS remaining. This is instead of the weak intermolecular bonding CF(H_2_O, N_2_, O_2_)PPS of untreated samples. Since HLEBI doses above 10 kGy appear to degrade the composite, carefulness is always recommended in adjusting for optimum HLEBI dose for practical applications.
(5) Specific future plans for this research are proprietary. However, in general, included are investigating specimens with different geometries, materials, tests, or treatments.

## Figures and Tables

**Table 1:** Charpy impact strength, *a*_uc_ (kJ m^−2^) and *P*_f_ for individual specimens from the data in Figure 5.

*P*_f_	*a*_uc_ (kJ m^−2^)				
	Unt’d	5 kGy	10 kGy	20 kGy	30 kGy
0.07	13.1	20.1	14.7	13.0	8.8
0.18	16.9	20.5	14.7	18.2	11.7
0.29	18.3	20.8	17.7	18.3	15.1
0.39	18.3	23.3	19.8	19.3	15.4
0.50	20.7	23.3	20.8	19.5	16.3
0.61	23.8	25.9	21.3	20.0	17.6
0.71	24.1	26.5	22.8	20.3	18.8
0.82	24.3	26.5	23.8	20.5	19.6
0.93	24.5	27.6	24.3	22.5	21.3

**Table 2:** Summary of the effect of the HLEBI dose on ESR signal intensities in PPS and *a*_uc_ at *P*_f_ = 0.50 of the [PPS]_4_[CF]_3_ composite. BDE values of AR-S and AR-H (kJmol^−1^) from [21,22] are shown.

HLEBI Dose (kGy)	AR-S Peak 1 ~285 kJmol^−1^	AR-H Peaks 2 and 3 ~461 kJmol^−1^	*a*_uc_ at *P*_f_ = 0.50 (kJ m^−2^)
0	YES	NO	20.7
5	<UNTREATED	NO	23.3
10	>>UNTREATED	YES	20.8
15	>UNTREATED	YES	-
20	>UNTREATED	YES	19.5

## Data Availability

Data are available upon request from the corresponding author. There was no obligation to make the data publicly available during the course of this project.
